# Humid heat waves at different warming levels

**DOI:** 10.1038/s41598-017-07536-7

**Published:** 2017-08-07

**Authors:** Simone Russo, Jana Sillmann, Andreas Sterl

**Affiliations:** 1 European Commission, Joint Research Centre, Ispra, Italy; 2 Institute for Environmental Protection and Research (ISPRA), Rome, Italy; 3 Center for International Climate and Environmental Research (CICERO), Pb. 1129 Blindern, N-0318 Oslo, Norway; 4 Royal Netherlands Meteorological Institute (KNMI), De Bilt, Netherlands

## Abstract

The co-occurrence of consecutive hot and humid days during a heat wave can strongly affect human health. Here, we quantify humid heat wave hazard in the recent past and at different levels of global warming. We find that the magnitude and apparent temperature peak of heat waves, such as the ones observed in Chicago in 1995 and China in 2003, have been strongly amplified by humidity. Climate model projections suggest that the percentage of area where heat wave magnitude and peak are amplified by humidity increases with increasing warming levels. Considering the effect of humidity at 1.5° and 2° global warming, highly populated regions, such as the Eastern US and China, could experience heat waves with magnitude greater than the one in Russia in 2010 (the most severe of the present era). The apparent temperature peak during such humid-heat waves can be greater than 55 °C. According to the US Weather Service, at this temperature humans are very likely to suffer from heat strokes. Humid-heat waves with these conditions were never exceeded in the present climate, but are expected to occur every other year at 4° global warming. This calls for respective adaptation measures in some key regions of the world along with international climate change mitigation efforts.

## Introduction

High temperatures and heat waves are known for their impacts on human health, for instance reflected in higher mortality rates^1^. The impact of heat waves can be due to a single variable, such as extreme temperature, or to a combination of variables not all of which are necessarily extreme^2^. In humid regions, the estimation of heat wave magnitude based only on temperature may underestimate the severity of a heat wave, as high humidity during consecutive hot days can be a contributory factor to amplify the effect of extreme heat waves^3–5^. In climatology, the occurrence of at least three consecutive hot days has been defined as a heat wave^6–9^. Here a heat wave is considered humid if at least one of its days shows an Apparent Temperature^10^ (hereafter AT, often referred to as heat index) that exceeds the dry-bulb temperature (T, in this case daily maximum temperature, see method). The AT is used to take into account the effect of relative humidity during hot days. It represents a non-linear measure of temperature and relative humidity^11^, which are generally negatively correlated^12, 13^, (Supplementary Fig. S1). For physiological stress, relative humidity is relevant because it describes the ability to lose heat through evaporative cooling^11, 14^

To take into account the effect of relative humidity during consecutive hot days, we introduce the new Apparent Heat Wave Index (AHWI), which is calculated analogously to the Heat Wave Magnitude Index daily HWMId^15^, but with daily maximum temperature replaced by AT for those heat wave days with *AT* > *T* (see method). We use two reanalysis sets (ERA-Interim^16^ and NCEP-2^17^) to compare the new AHWI, calculated with daily maximum temperature and daily minimum relative humidity (see method), with the existing HWMId^15^, which is based on temperature only.

Additionally, by using daily maximum temperature and minimum relative humidity outputs from the Coupled Model intercomparison Project Phase 5 (CMIP5^18^), we project heat wave and apparent heat wave magnitudes at different warming levels (1.5°, 2° and 4°) relative to the period 1861–1880 (see method). In order to show the potential impact of the combination of temperature and relative humidity on human health during a heat wave, we consider AT peak (*AT*_*peak*_) defined as the maximum AT value during a heat wave (see method).

We analyze changes in the yearly probability of *AT*_*peak*_ higher than 40 °C (~105°F, hereafter AT40C) and 55 °C (~130° F, hereafter AT55C). These thresholds are the ones at which the US National Weather Service issues a heat advisory because of dangerous health conditions (high incidence of heat cramps, heat exhaustion and heat strokes) and very dangerous health conditions (heat stroke very likely), respectively.

## Results

The spatial distribution of the 90^*th*^ percentile of the HWMId and AHWI calculated at each grid point within the period 1979–2015 compares well between reanalysis (ERA-Interim and NCEP-2) and model data (see Supplementary Figs S2 and S3). Additionally, the HWMId, AHWI, and AT40C spatial patterns compare remarkably well across models (Supplementary Figs S4, S5 and S6, respectively) and strongly resemble the reanalysis (Supplementary Figs. S2a,b,d,e and S3a,b) and ensemble models patterns (Supplementary Figs S2c,f and S3c). These results confirm those of a recent study^19^ showing robust agreement in heat stress variables (based on temperature and relative humidity) in a set of CMIP5 models.

**Figure 1:**
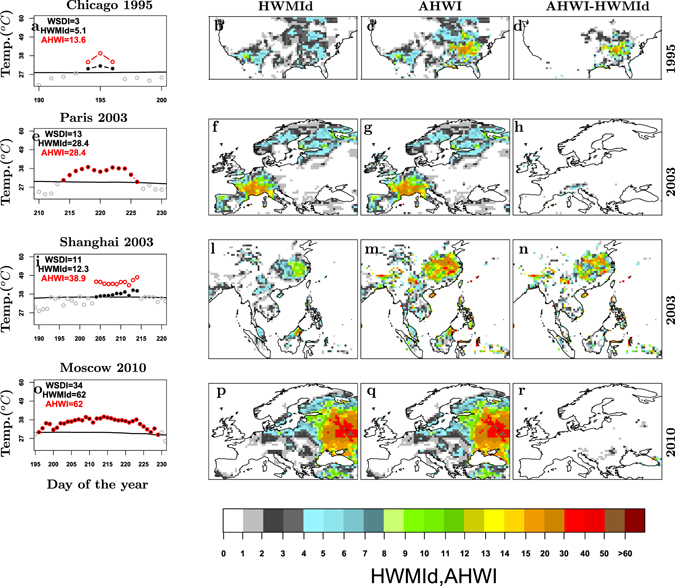
Historical heat waves and humid heat waves across world regions with different climates and their HWMId and AHWI spatial distribution. The panels (**a**,**e**,**i**,**o**) show time series of heat waves that occurred in specific locations reporting the values of HWMId, AHWI and the duration, calculated by means of the Warm Spell Duration Index (WSDI, see method). Black line is the 90^*th*^ percentile daily threshold (see method). Open grey circles are the temperature values of the days. ﻿Full black circles﻿ are the days in the heat wave. Red open circles represent the apparent temperature values calculated, for each day composing the heat wave, by means of the Heat Index (see method). The maps in (**b**,**f**,**l**,**p**) and (**c**,**g**,**m**,**q**) represent the spatial distribution of the HWMId and AHWI, respectively. The maps in (**d**,**h**,**n**,**r**) show the spatial distribution of the differences between AHWI and HWMId. This figure has been produced using R version 3.3.2 (https://cran.r-project.org/).

In some key regions, such as the Midwestern and Eastern US, China, Northern Latin America and Malaysia, both reanalysis and model data show that heat wave magnitude and peak temperature have been amplified by high relative humidity in the recent past. The selected heat waves of Chicago 1995 and Shanghai 2003 are clear examples of the contribution of relative humidity during a heat wave (Fig. 1a–d,i–n). On the contrary, the effect of relative humidity was negligible during the two famous heat waves that occurred in Central Europe in 2003 and in Russia in 2010 (Fig. 1e–h,o–r).

In both humid and dry regions heat wave occurrence can be associated with persistent synoptic conditions such as: blocking patterns, reduced cloudiness and advection of warm air^20, 21^. However, in regions such as the Eastern US and China, where apparent temperature impacts are amplified by high relative humidity, the formation of a humid heat wave typically is due to hot and humid air being advected from the Gulf of Mexico or from tropical regions^22^, respectively. In contrast, across Europe and the Western US, the formation of a dry heat wave is often due to a blocking pattern and associated advection of hot dry air from desert areas. As an example the 2010 Russian heat wave resulted from a strong blocking anticyclone driving warm air from Africa^23^.

During the heat wave of 1995, Chicago was referred to as “the Urban Inferno^24^”. The heat wave had a high impact because extreme temperatures were made worse by high relative humidity, which made it difficult to sweat and cool off. From a physiological point of view, this short heat wave would not have been so extreme without high relative humidity^25, 26^ (Fig. 1a–d).

In the summer of 2003, two heat waves hit Europe^21^ and China^27^ (Fig. 1e–h,i–n, respectively). By comparing these two events, we see that the heat wave across central Europe was mainly due to extreme temperatures, whereas during the hottest summer of the past 50 years recorded in Shanghai in 2003 high relative humidity played an important role in exacerbating the heat wave impact^27^ (Fig. 1i–n).

Another example of extreme but not humid heat wave is represented by the heat wave that occurred in Russia in the summer of the 2010 (Fig. 1o–r). This heat wave is considered the most extreme one of the present era^15, 21^. For this heat wave the maximum magnitude exceeded the score of 60 (Fig. 1o–r), a value that, according to all our datasets, was never recorded during any other heat wave of the recent past period (1979–2015). Additionally, the HWMId and AHWI values calculated for the same recent past period from the CMIP5 models do not show any location exceeding a magnitude of 60. This threshold is used as reference to estimate the probability of occurrence of an extreme heat wave with magnitude greater than the one in Russia in 2010 (hereafter, RU2010).

**Figure 2:**
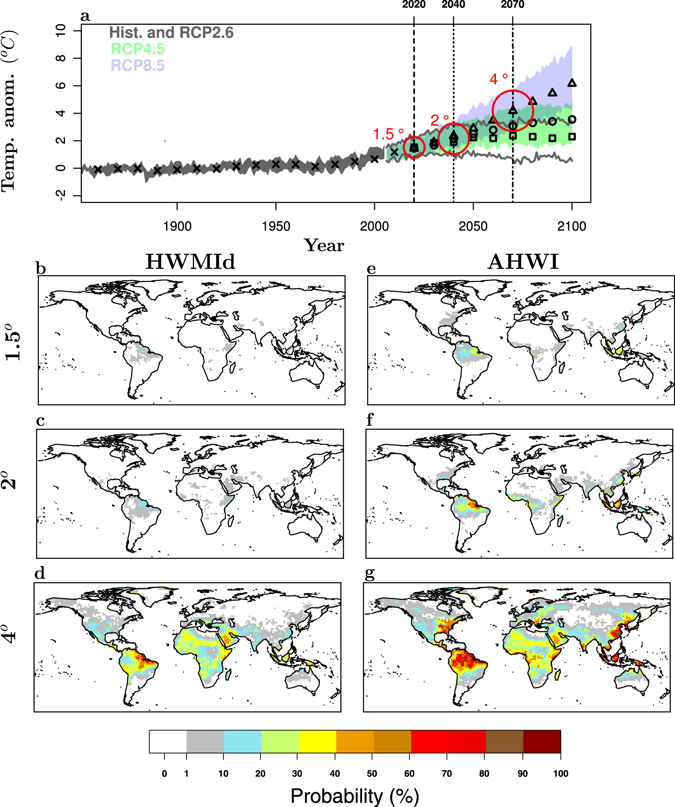
Probability of occurrence of extreme humid heat waves at different warming levels relative to 1861–1880. (**a**), Simulated global mean surface temperature increase as a function of time. Decadal model median over the historical period (1860–2010) are represented by black crosses. Decadal model median over the future period (2011–2100) for the three Representative Concentration Pathway (RCP2.6, RCP4.5 and RCP8.5) scenarios are represented by black squares, circle and triangles, respectively. (**b**–**d**), Probability of occurrence of heat waves with magnitude greater than the maximum magnitude detected in Russia in 2010 (HWMId > 60) calculated at each grid point for all model years with global mean temperature anomaly relative to 1861–1880 between 1.4° and 1.6° (1.5° warming level, see method), 1.9°–2.1° (2° warming level) and 3.9°–4.1° (4° warming level), respectively. e-g, as b-d but for humid heat waves and the relative Apparent Heat Wave Index (AHWI > 60). This figure has been produced using R version 3.3.2 (https://cran.r-project.org/).

In the coming decades, with increasing global mean temperature, the global land fraction with high probability of occurrence of humid heat waves with magnitude greater than RU2010 is expected to increase (Fig. 2). On a global scale, the near-surface *absolute humidity* of the air roughly follows the increasing temperatures according to the Clausius–Clapeyron relationship, and thus *relative humidity* remains roughly constant or slightly decreases over land^28^. However, locally the relative humidity may change due to circulation changes^28^. Moreover, the non-linear terms in the definition of AT amplify the impact of temperature changes even if relative humidity remains constant. The humidity-induced heat stress amplification is strongest in the regions that are warmest and most humid under present-day conditions^19, 29^ Our results are consistent with earlier studies showing that heat stress is projected to increase over all land regions along with rising temperatures^19^.

At 1.5 °C and 2 °C warming above pre-industrial levels, the probability of occurrence of a RU2010 heat wave is zero almost everywhere if measured with the HWMId (Fig. 2b,c). On the contrary, this probability is different than zero, although still very small, in the Eastern US, China, Central West Africa and Northern Latin America when measured by means of the AHWI, which takes into account both temperature and relative humidity (Fig. 2e,f). At 4 °C warming the yearly probability of occurrence of a heat wave with magnitude greater than the RU2010 will be greater than 10% in Central Europe, India, and across many African regions. The Eastern US, Northern Latin America and China are expected to experience such type of heat waves with a annual probability greater than 50%, corresponding to an average return period of two years (Fig. 2g). This probability is greater than the one projected in the hottest world regions, such as the Arabian Peninsula, Australia and other dry-deserts (see Fig. 2d,g).

**Figure 3:**
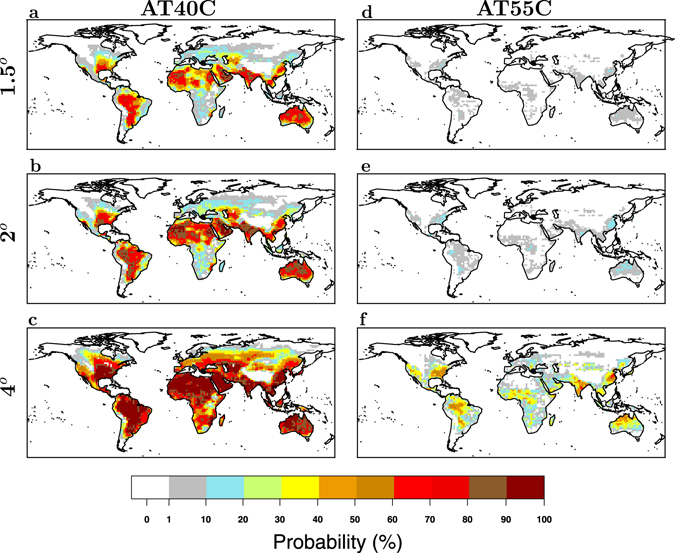
Annual probability of occurrence of a heat waves with apparent temperature peaks greater than 40 °C and 55 °C. (**a**–**c**), Probability of occurrence of heat waves with *AT*_*peak*_ ≥ 40 (AT40C) calculated at each grid point for all model years with global mean temperature anomaly relative to 1861–1880 at 1.5, 2, and 4 degrees warming (see Fig. 2), respectively. (**d**–**f**), as (**a**–**c**) but for occurrence of heat waves with *AT*_*peak*_ ≥ 55 (AT55C). This figure has been produced using R version 3.3.2 (https://cran.r-project.org/).

Similar results are found for the occurrence of heat waves with AT40C and AT55C peaks. The percentage of global land area with a high probability of occurrence of heat waves with AT40C and AT55C increases with increasing warming levels (Fig. 3). Across the hottest world regions and the regions where relative humidity amplifies the heat wave magnitude, the probability of annual occurrence of a heat wave with a AT40C peak show annual values exceeding 50% and 90% at a warming level of 2 °C and 4 °C, respectively (Fig. 3b,c). These probability values are much smaller if measured only with temperature (see supplementary Fig. S7a–c). Highly populated regions, such as the Eastern US and China, are expected to experience the warmest *AT*_*peak*_ values of the world (see Supplementary Fig. S8), with an occurrence of a AT55C peak on a two year basis (probability greater than 50%, see Fig. 3f). Due to the high expected apparent temperature values these regions are projected to have high number of deaths among people older than 65 years in 2050^30^, without assuming adaptation effects. Note that, as for the heat wave magnitude across these regions, all warming levels show a probability of occurrence of AT55C equal to or greater than the one of the warmest world regions such as dry-deserts (Fig. 3d–f). Without considering relative humidity, the probability that heat wave temperature peaks exceed 55 °C is equal or very close to zero almost everywhere at all warming levels (see Fig. S7d–f).

The occurrence of heat waves with AT55C, never recorded in our data records in the recent past, is likely to cause heat strokes by limiting the human thermoregulation. The exceedance of this apparent temperature across these regions is in agreement with other measures accounting for the combined effect of temperature and relative humidity. As an example, the wet-bulb temperature peak during a heat wave is expected to exceed the value of 35 °C (see Supplementary Fig. S9), a threshold likely to induce hyperthermia in humans and other mammals as dissipation of metabolic heat becomes impossible^25, 31^ While this never happens in the present climate, and it is unlikely at 1.5 °C and 2 °C, it would occur on a regular basis in many highly populated regions with global-mean warming of about 4 °C, questioning the habitability of some of these regions.

## Discussion

Our results show that some of the most densely populated regions are among those that are most exposed to humid heat waves. In the recent past, the severity of heat waves across urban areas, such as Chicago and Shanghai, that are considered non-severe from a temperature-only point of view, was strongly increased when considering relative humidity. Due to the humidity effect the AT_*peak*_ values across these cities are projected to reach extremely severe values in the future with the rising of global mean temperature. At 4 °C global warming, the apparent heat wave magnitude is greater than the highest present value, with *AT*_*peak*_ exceeding the level of 55 °C, (critical for human survival) at least once in two years. The effect of relative humidity on the heat wave magnitude and peak might even be underestimated here. This is because the AT is calculated by using daily minimum relative humidity instead of relative humidity occurring at the time of the daily maximum temperature, which is not available in the current models. Further, this study is on heat wave hazards and does not take into account the effects of adaptation or other socio-economic factors^32, 33^, (for example, improved housing or access to air-conditioning). Our results give an indication of where relative humidity might be an important amplifying factor for heat stress. Changes in heat wave hazard are very important and informative, indicating regions where adaptation measures might be necessary to cope with heat stress.

## Methods

Daily maximum temperature and minimum relative humidity data from the ERA-Interim (ERA-I) reanalysis from the European Centre for Medium-Range Weather Forecasts^16^, the NCEP-2 reanalysis from the National Centers for Environmental Prediction-Department of Energy, and from nine climate model simulations contributing to CMIP5^18^ (see Supplementary Table 1) are used to study heat waves in the present and future climate. The selection of these nine CMIP5 models is based on the availability of daily maximum temperature and minimum relative humidity data. Reanalysis data have a 6-hourly time resolution and are available from 1979 onward. The ERA-I is based on a T255 resolution (~79 km) grid. For comparison the data are bilinearly interpolated onto the NCEP-2 grid, which has a resolution of 1.875 degrees (~208 km). Similarly, daily maximum temperature and minimum humidity from climate model output were remapped to a common 1.875° × 1.875° analysis grid (T63) to allow comparison with the reanalysis.

We calculate the Heat Wave Magnitude Index daily^15^ (HWMId), which depends only on temperature, and the new Apparent Heat Wave Index (AHWI), which is based on temperature and humidity. HWMId was calculated for the historical scenario^18^ from 1850 to 2005, and for three Representative Concentration Pathway scenarios^18^ (RCP2.6, RCP4.5 and RCP8.5) from 2006 to 2100. The calculation of the AHWI has been done for the same periods and scenarios as for the HWMId, with the exception that for the historical period we started in 1970, since the humidity data were not available for all the used models before this year. We use one run (r1i1p1) for each model. Global mean temperatures (in Fig. 2a) were calculated as the area-weighted global averages of annual mean temperatures.

### Heat Wave Magnitude Index daily

The HWMId is defined as the maximum magnitude of heat waves in a year^15^, where a heat wave is a period of at least 3 consecutive days with temperature exceeding the daily threshold for the reference period (1981–2010). The threshold is defined as the 90th percentile of daily temperatures, centered on a 31 day window. The magnitude of each heat wave in a year is the sum of the magnitude of the consecutive days composing a heat wave, with daily magnitude calculated as follow:

1 $${M}_{d}({T}_{d})=\{\begin{array}{cc}\frac{{T}_{d}-{T}_{30y25p}}{{T}_{30y75p}-{T}_{30y25p}} & {\rm{i}}{\rm{f}}\,{T}_{d} > {T}_{30y25p}\\ 0 & {\rm{if}}\,{T}_{d}\le {T}_{30y25p}\end{array}$$

with *T*_*d*_ being the daily maximum temperature on day d of the heatwave, *T*_30*y*25*p*_ and *T*_30*y*75*p*_ are the 25^*th*^ and 75^*th*^ percentile values, respectively, of the time series composed of 30-year annual maximum temperatures within the reference period 1981–2010. The HWMId calculation has been done by using the “hwmid” R function^34^.

### Warm Spell Duration Index

The Warm Spell Duration Index, defined by the Expert Team on Climate Change Detection and Indices (ETCCDI) (http://etccdi.pacificclimate.org/list_27_indices.shtml), counts the number of consecutive days of a heat wave (at least 3 consecutive days) with *T*_*d*_ > 90^*th*^ percentile daily threshold defined above.

### Apparent Temperature

The apparent temperature, often referred to as heat index (HI) combines air temperature and relative humidity in order to determine the human-perceived equivalent temperature^10^. There are many different versions of apparent temperature. Here we apply the one used by NOAA (see http://www.wpc.ncep.noaa.gov/html/heatindex_equation.shtml):

2 $$AT={c}_{1}+{c}_{2}T+{c}_{3}R+{c}_{4}TR+{c}_{5}{T}^{2}+{c}_{6}{R}^{2}+{c}_{7}{T}^{2}R+{c}_{8}T{R}^{2}+{c}_{9}{T}^{2}{R}^{2}$$

where:

AT is the apparent temperature in degrees Fahrenheit °F;

T is temperature (°F);

R is the relative humidity between 0 and 100;

*c*_1_ = −42.379; c_2_ = 2.04901523; *c*_3_ = 10.14333127; *c*_4_ = −0.22475541; *c*_5_ = −6.83783 × 10^−3^; *c*_6_ = −5.481717 × 10^−2^; *c*_7_ = 1.22874 × 10^−3^; *c*_8_ = 8.5282 × 10^−4^; *c*_9_ = −0.199 × 10^−6^.

The AT values are transformed in Celsius after calculation. Additionally, for all the temperature range where the AT does not work properly we have applied all the adjustments reported in the fact sheet of the NOAA’s web page (http://www.wpc.ncep.noaa.gov/html/heatindex.equation.shtml).

### Apparent Heat Wave Index

The Apparent Heat Wave Index is defined analogously to the HWMId, but by replacing the T_d_ temperature in equation (1) by the apparent temperature as given (equation (2)) for days on which AT > T. The AHWI and the previous HWMId are comparable. A AHWI score twice as large as the one of the HWMId means that the heat wave magnitude is amplified twice by humidity. Ideally the AT and the AHWI should be calculated form the daily maximum temperature and simultaneous relative humidity. As the latter is not available for the CMIP5 models we use the daily minimum relative humidity, as done in other studies^11^, which at first approximation coincides with the maximum temperature at the diurnal cycle. Note that, in order to remove the bias, consisting in a discontinuity between estimation of the two indicators within the reference period (1981–2010) and the ones out-of-base periods^35, 36^, the HWI and HWMId in the base period have been calculated by means of a bootstrap re-sampling procedure^35^.

### Apparent Temperature peak

The apparent temperature peak (AT_*peak*_) is defined as the maximum AT value during a heat wave. For each specific year we calculate the maximum apparent temperature value. From the AT_*peak*_ we define two binary variables: AT40C and AT55C. For each year the AT40C and AT55C are equal to one if the AT_*peak*_ > 40 °C and AT_*peak*_ > 55 °C, respectively. Otherwise they are zero.

### Estimation of empirical probability

The yearly probability of occurrence of a heat wave with a peak greater than a certain temperature (40 °C and 55 °C) and a magnitude greater than 60 (recorded only in Russia in 2010) has been estimated for the recent past-period (1979–2015) and at different warming levels (1.5 °C, 2 °C, and 4 °C) by using empirical probability. The empirical probability or empirical frequency^37^ of an event is defined as the ratio between the number of outcomes in which a specified event occurs (e.g. number of years of the recent past-period with *AT*_*peak*_ > 40 °C) and the total number of trials (e.g. total number of years of the recent past period (1979–2015)). The sample size of the set composed by model years with global mean temperature at 1.5 °C, 2 °C, and 4 °C warming has been estimated equal to 115, 115 and 116, respectively.

## Electronic supplementary material

Supplementary Information
