# Supplementary material for: Humid heat waves at different warming levels
Source: Sci Rep. 2017 Aug 7;7:7477. doi: 10.1038/s41598-017-07536-7 (PMC5547064; doi:10.1038/s41598-017-07536-7)
Supplement: Supplementary file 1 — Supplementary Information [file 41598_2017_7536_MOESM1_ESM.pdf]

Humid heat waves at different warming levels-Supplementary Information

Simone Russo[1,2,*], Jana Sillmann[3], Andreas Sterl[4]

June 12, 2017

1. European Commission, Joint Research Centre, Ispra, Italy

2. Institute for Environmental Protection and Research (ISPRA), Rome, Italy

3. Center for International Climate and Environmental Research (CICERO), Pb. 1129 Blindern, N-0318 Oslo, Norway.

4. Royal Netherlands Meteorological Institute (KNMI), De Bilt, Netherlands.

Model	Institution	Spatial Resolution (Lon×Lat)
1 CNRM-CM5	Centre National de Recherches Meteorogiques, Meteo France, France	256×128(T85)<br>128×64(T42)
2 GFDL-ESM2G	Geophysical Fluid Dynamics Laboratory, USA	144×90
3 GFDL-ESM2M	Geophysical Fluid Dynamics Laboratory, USA	144×90
4 IPSL-CM5A-LR	Institut Pierre-Simon Laplace, France	96×96(T42)
5 IPSL-CM5A-MR	Institut Pierre-Simon Laplace, France	144×143(T42)
6 MIROC5	AORI (Atmosphere and Ocean Research Institute), NIES (National Institute for Environmental Studies), JAMSTEC (Japan Agency for Marine-Earth Science and Technology), Japan	256×128(T85)
7 MIROC-ESM	AORI, NIES, JAMSTEC, Japan	128×64(T42)
8 MIROC-ESM-CHEM	AORI, NIES, JAMSTEC, Japan	128×64(T42)
9 MRI-CGCM3	Meteorological Research Institute, Japan	320×160(T106)

Supplementary Table 1 — List of the AR5 CMIP5 used models.

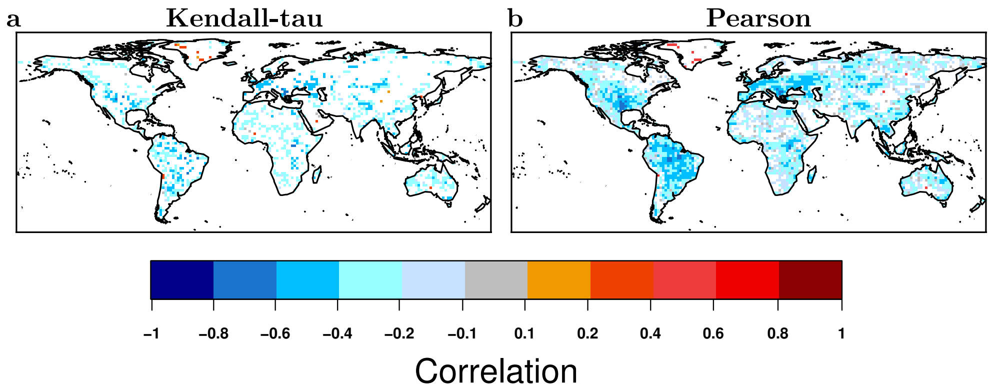

Supplementary Figure 1 — Correlation between temperature and humidity Kendall-tau and Pearson correlation estimates between annual maximum temperature and simultaneous relative humidity within the period 1979-2015 with ERA-Interim data. White areas represent locations at which correlation is not significant (p-value>10%). This figure has been produced using R version 3.3.2 (https://cran.r-project.org/).

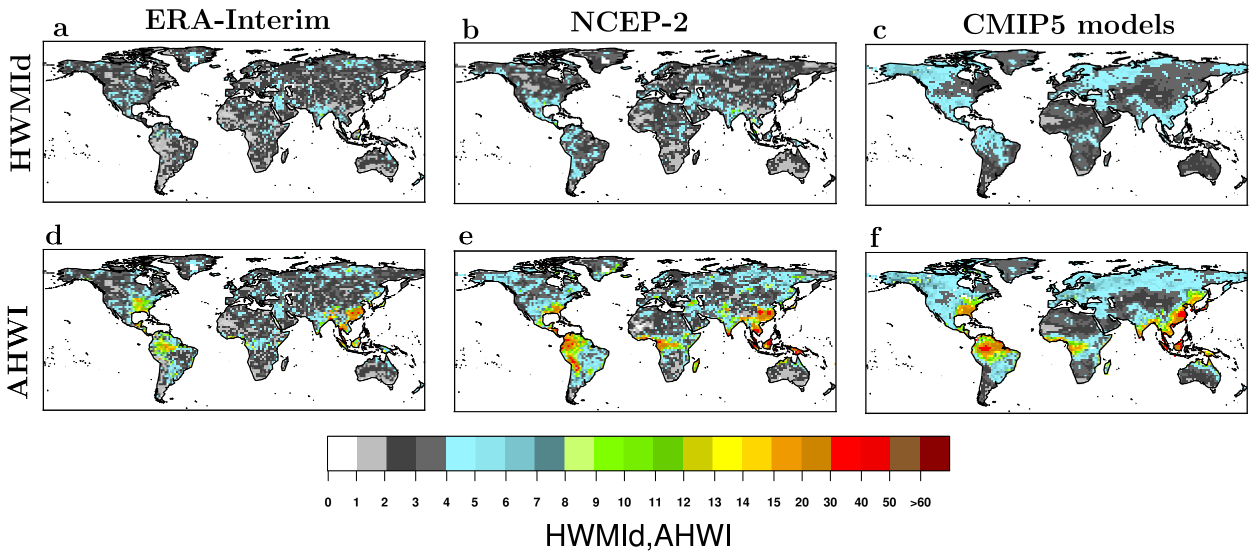

Supplementary Figure 2 — Spatial distribution of the 90th HWMId and AHWI percentile. The values are estimated at each specific grid point for the recent past period 1979-2015. a-c 90th HWMId percentile calculated with ERA-Interim, NCEP-2, and the ensemble of 9 models, respectively. d-f, as a-c but for Apparent Heat Wave Index (AHWI). This figure has been produced using R version 3.3.2 (https://cran.r-project.org/).

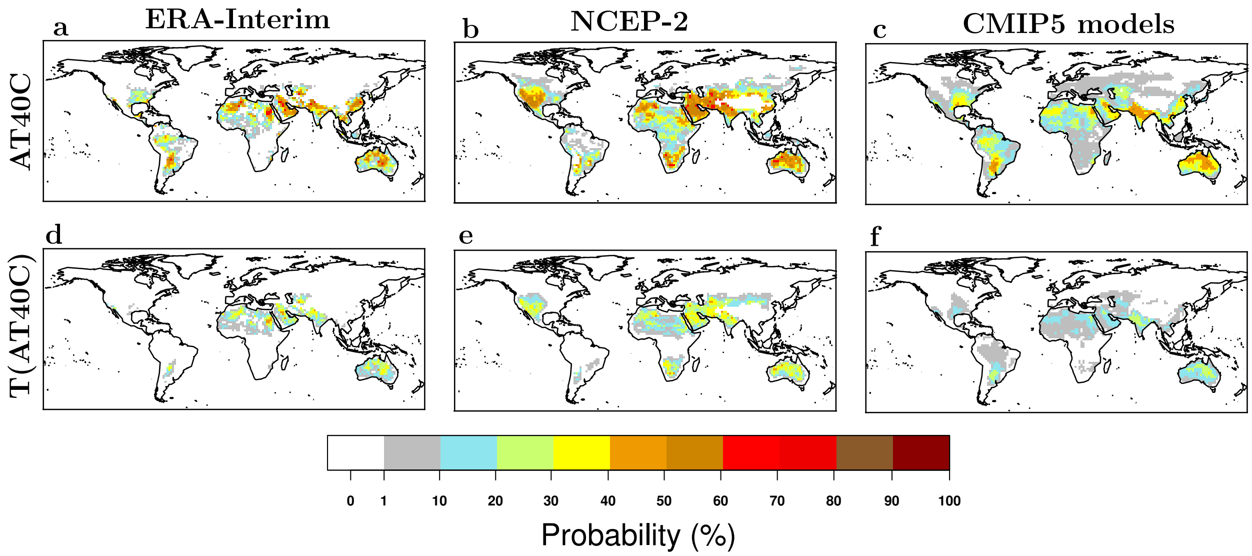

Supplementary Figure 3 — Annual probability of occurrence of heat wave with both AT_{peak} and corresponding temperature T equal to or greater than 40^oC. Probability values are estimated at each specific grid point in the period 1979-2015 for reanalysis and CMIP5 model data (see methods). Note that reanalysis and models data show reasonable agreement in key regions such the Eastern US and China where high humidity combined with annual maximum temperature amplifies the heat wave peak temperature. In fact, while AT40C show probability greater than 10% (a-c), the temperature associated to the AT40C (T(AT40C)) show zero probability to exceed 40^oC during a heat wave across these regions (d-f). This figure has been produced using R version 3.3.2 (https://cran.r-project.org/).

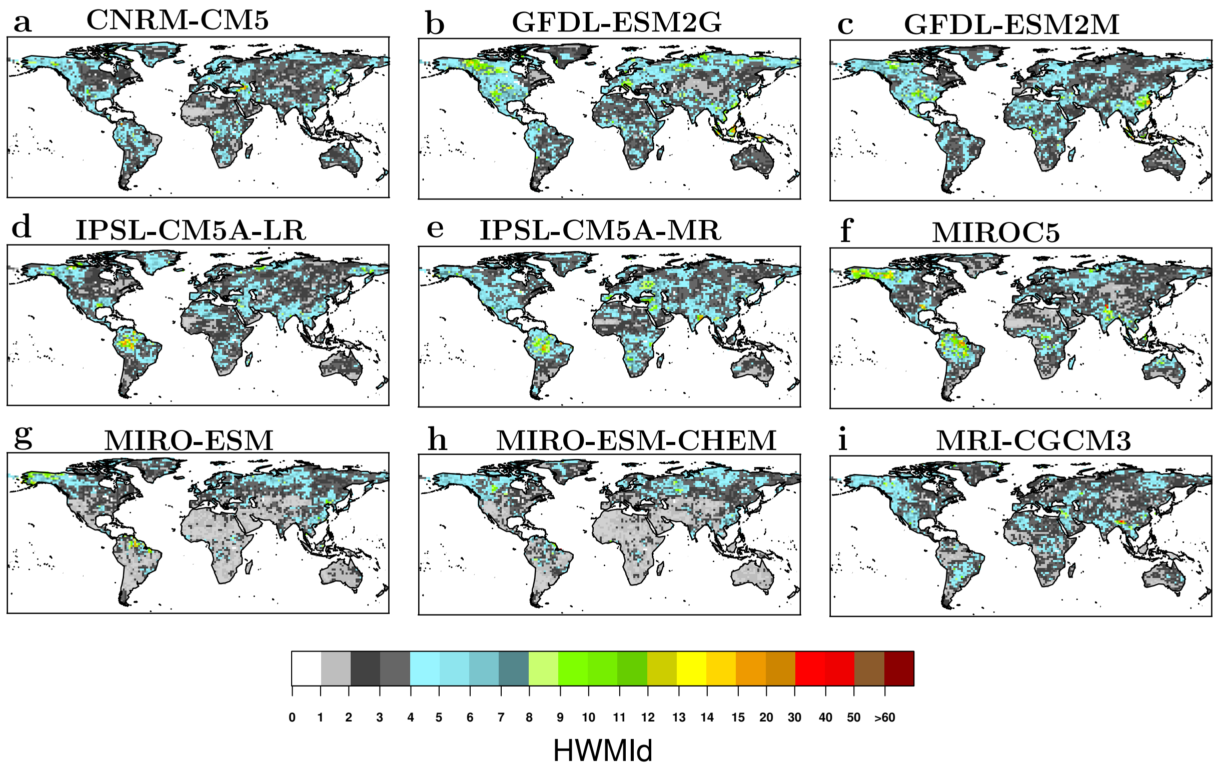

Supplementary Figure 4 — Spatial distribution of the 90th HWMId percentile for each single model. The values are estimated at each specific grid point for the recent past period 1979-2015. This figure has been produced using R version 3.3.2 (https://cran.r-project.org/).

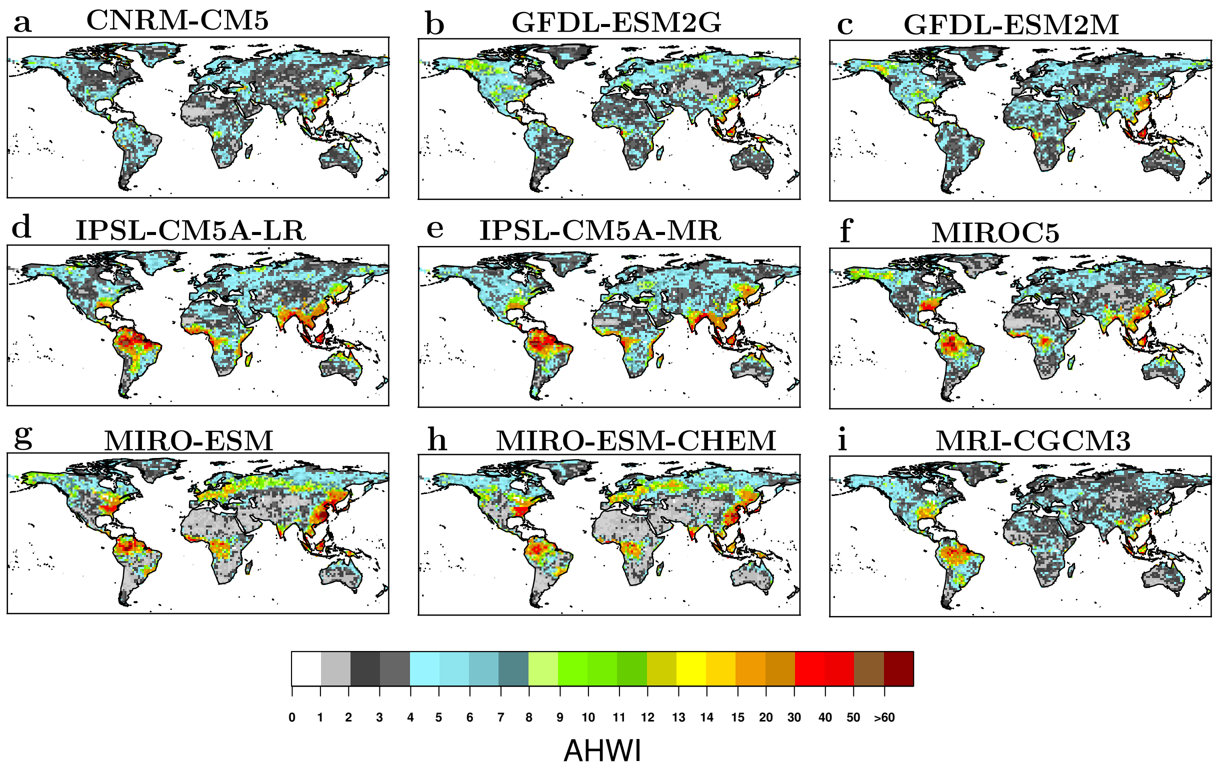

Supplementary Figure 5 — Spatial distribution of the 90th AHWI percentile for each single model. The values are estimated at each specific grid point for the recent past period 1979-2015. This figure has been performed using R version 3.3.2 (https://cran.r-project.org/). This figure has been produced using R version 3.3.2 (https://cran.r-project.org/).

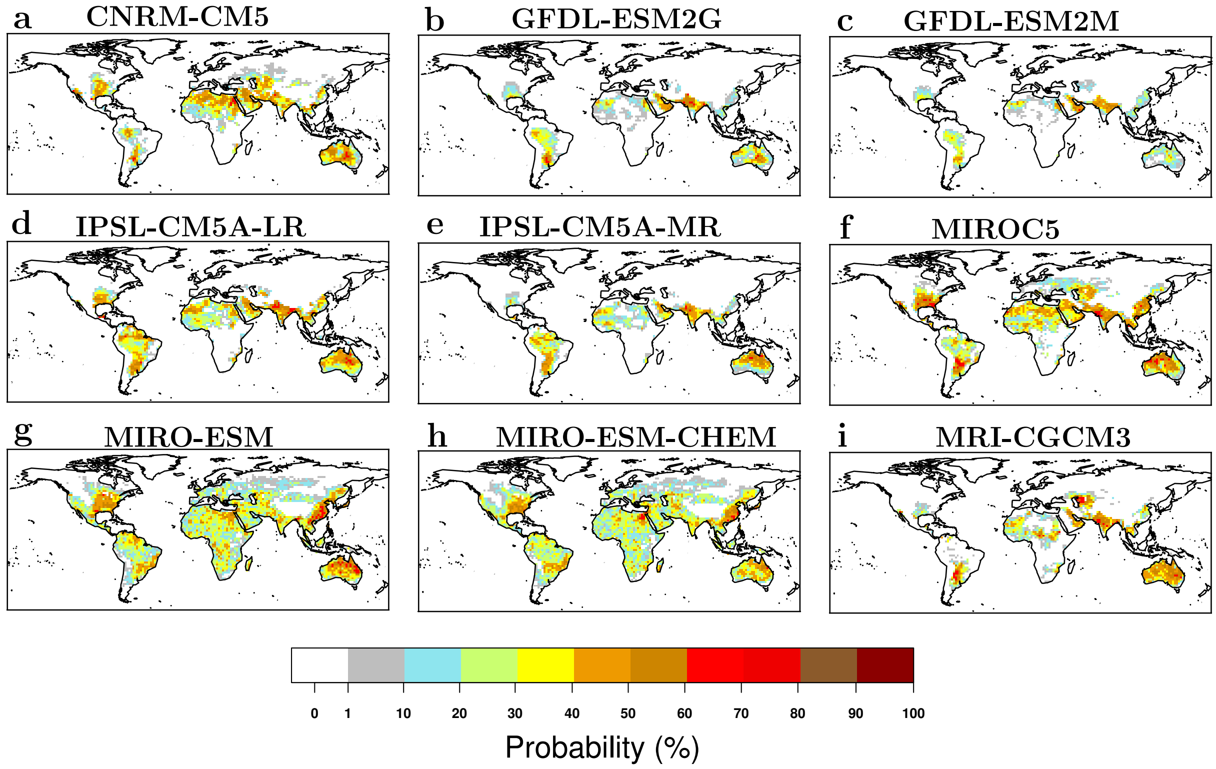

Supplementary Figure 6 — Annual probability of occurrence of heat wave with both AT_{peak} and corresponding temperature AT equal to or greater than 40^oC. Probability values are estimated at each specific grid point in the period 1979-2015 for each CMIP5 model data (see methods). This figure has been produced using R version 3.3.2 (https://cran.r-project.org/).

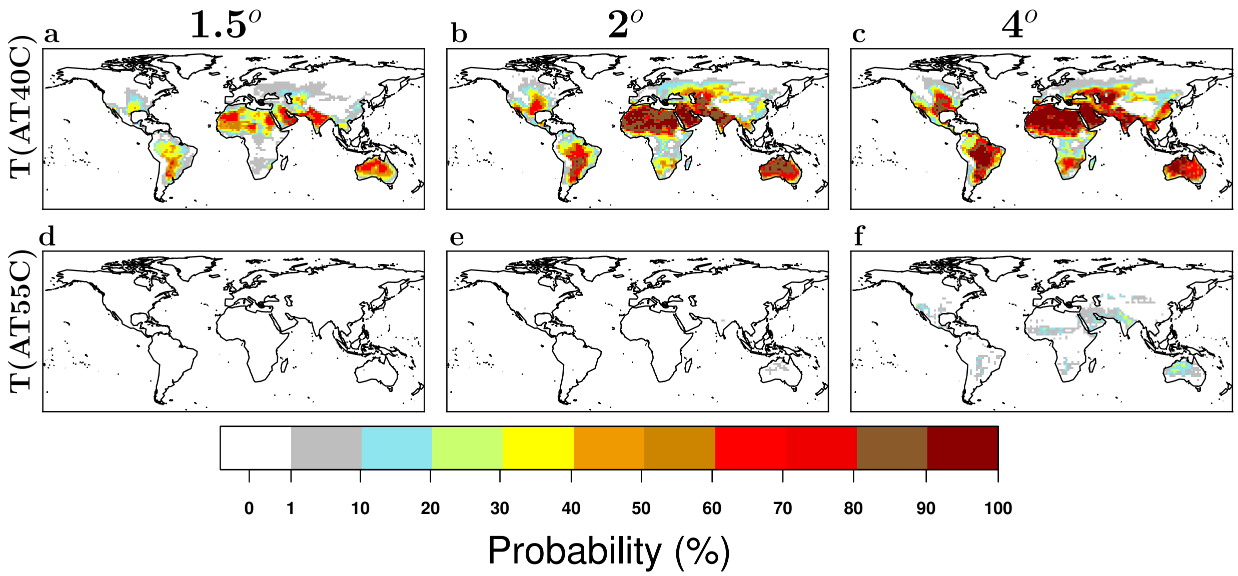

Supplementary Figure 7 — Annual probability of occurrence of heat wave with temperature T (corresponding to apparent temperature) greater than 40^oC **and** 55^oC**.** Probability values are estimated at different warming levels relative to 1861-1880. a-c for $T > 40^oC$. d-f for $T > 55^oC$. This figure has been produced using R version 3.3.2 (https://cran.r-project.org/).

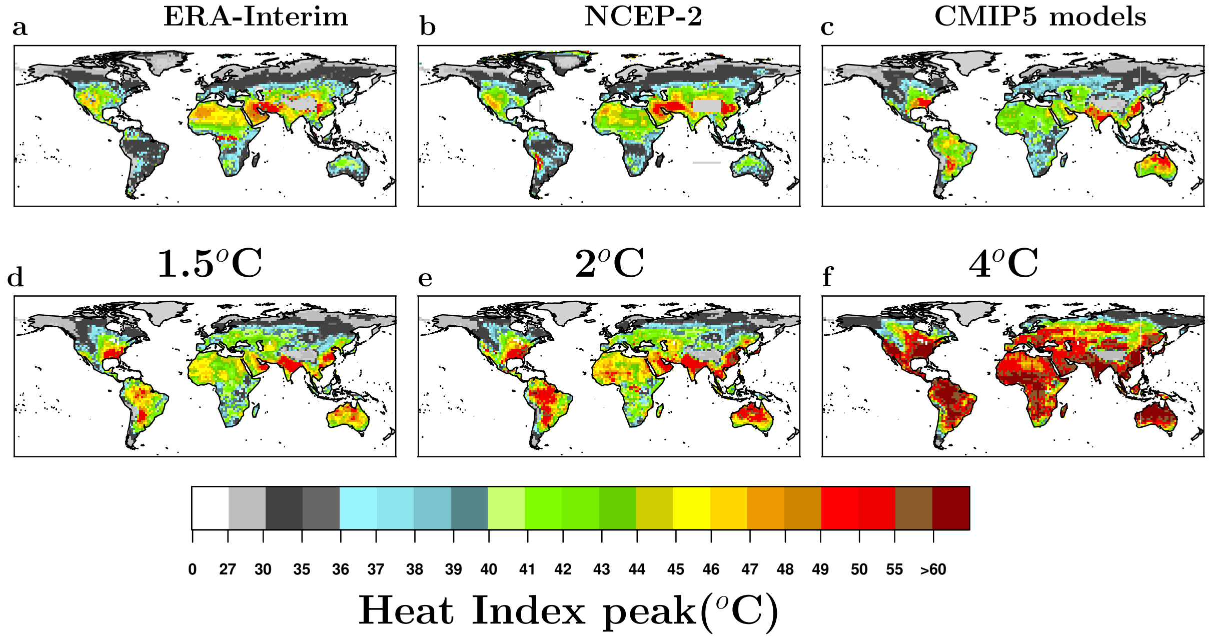

Supplementary Figure 8 — Spatial distribution of 90th percentile of apparent heat wave temperature peak. Values are estimated at each specific grid point within the period 1979-2015 with reanalysis and CMIP5 model data (see method). a-c, recent-past period (1979-2015) with ERA-Interim, NCEP-2 reanalysis and CMIP5 models, respectively. d-f, as a-c, but at 1.5^{o}C, 2°C and 4°C levels, respectively. This figure has been produced using R version 3.3.2 (https://cran.r-project.org/).

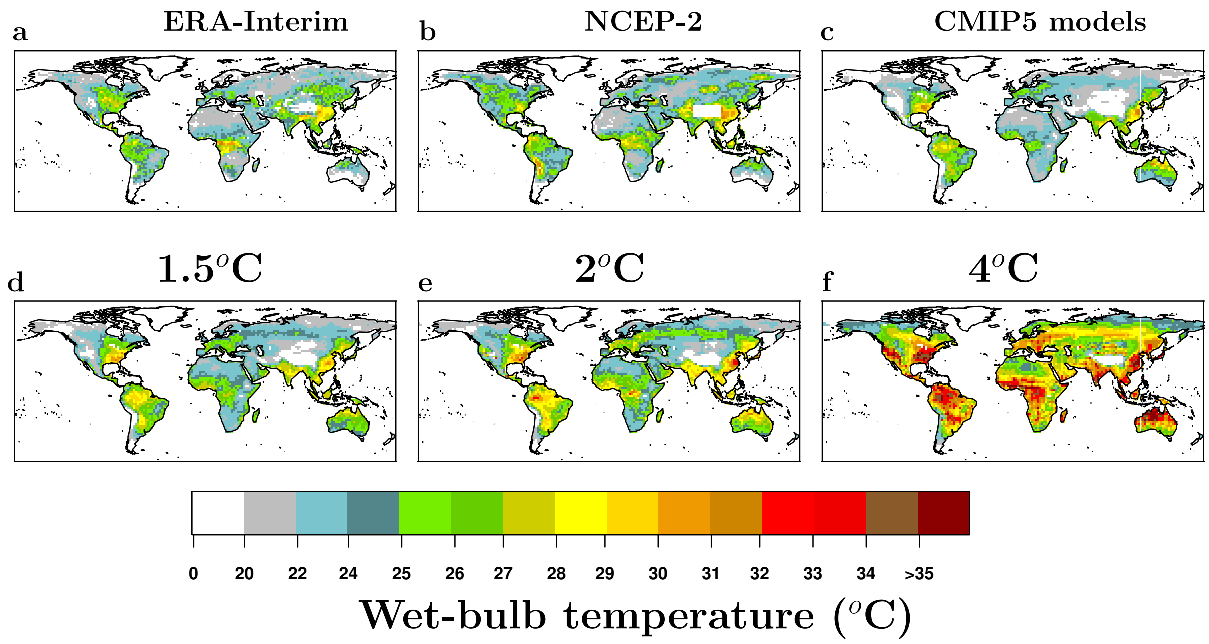

Supplementary Figure 9 —Spatial distribution of 90th percentile of the heat wave wet-bulb temperature peak. Values are estimated at each specific grid point within the period 1979-2015 with reanalysis and CMIP5 model data (see method). a-c, recent-past period (1979-2015) with ERA-Interim, NCEP-2 reanalysis and CMIP5 models, respectively. d-f, as a-c, but at 1.5^{o}C, 2°C and 4°C levels, respectively. This figure has been produced using R version 3.3.2 (https://cran.r-project.org/).
